# Sydney Smith as a Doctor

**Published:** 1887-04-09

**Authors:** 


### Sydney Smith as a Doctor.

(*The Times*, August 10-14, 1855.)

Whilst the great wit held the living of Foston-le-Clay in Yorkshire, "he was," says *The Times*, "ingeniously active on behalf of his neighbours and parishioners. Many a hungry labourer was brought in and stuffed with rice or broth or porridge, to test their relative effects on the appetite. He set on foot gardens for the poor, and became their doctor and general comforter. He played the part of village Æsculapius, in virtue of his former attendance at some medical lectures in Edinburgh. Several volumes of his prescriptions, it is said, remain; but, according to his own account, his practice was particularly simple. 'Where is Annie Kay? Ring the bell for Annie Kay.' Kay appeared. 'Bring me my medicine-book, Annie Kay. Kay is my apothecary's boy, and makes up my medicines.' Kay appears with the book. 'I am a great doctor; would you like to hear some of my medicines?' 'Oh yes, Mr. Sydney.' 'There is the *Gentle-jog*, a pleasure to take it; the *Bull-dog*, for more serious cases; *Peter's puke*, *Heart's delight*, the comfort of all the old women in the village; *Rub-a-dub*, a capital embrocation; *Dead-stop*, settles the matter at once; *Up-with-it-then*, needs no explanation; and so on. Now, Annie Kay, give Mrs. Spratt a bottle of *Rub-a-dub*, and to Mrs. Coles a dose of *Dead-stop* and twenty drops of laudanum.'

"'This is the house to be ill in (turning to us); indeed, everybody who comes is expected to take a little something. I consider it a delicate compliment when my guests have a slight illness here. We have contrivances for everything. Have you seen my patent armour? No? Annie Kay, bring my patent armour. Now, look here. If you have a stiff neck, or swelled face, here is this sweet case of tin filled with hot water, and covered with flannel, to put round your neck and you are well directly. Likewise a patent tin shoulder, in case of rheumatism. There you see a stomach-tin, the greatest comfort in life; and lastly, here is a tin slipper, to be filled with hot water, which you can sit with in the drawing-room, should you come in chilled, without wetting your feet. Come and see my apothecaries shop.'

"We all went downstairs, and entered a room filled entirely on one side with medicines, and on the other with every description of groceries and household or agricultural necessaries; in the centre a large chest forming a table, and divided into compartments for soap, candles, salt, and sugar.

"'Here you see,' said he, 'every human want before you—

'Man wants but little here below,
As beef, veal, mutton, pork, lamb, venison show.'

spreading out his arms to exhibit everything, and laughing. 'Life is a difficult thing in the country, I assure you, and it requires a good deal of forethought to steer the ship when you live twelve miles from a lemon.'

"All this forethought, however, could not prevent occasional misadventures. A horse named 'Peter the Cruel,' on one occasion ate, by his groom's mistake, instead of a 'ball,' two boxes of opium pills in his bran-mash, boxes and all. Another time he found all his pigs intoxicated, and, as he declared, grunting, 'God save the King,' about the sty, from having eaten some fermented grains, which he himself had ordered for them. *Per contra*, his medical skill sometimes availed to *repair* similar mishaps. He probably saved the life of his footman, who had eaten some dough prepared with arsenic for the rats, and which had been left on the kitcher-dresser. The man, we are told, had a passion for dough. 'He swallowed,' says Sydney, 'as much arsenic as would have poisoned all the rats in the House of Lords; but I pumped lime-water into him night and day for many hours at a time; and there he is.' *Jeames* was lucky in a master who enabled him to offer such evidence of his preservation.

"When he was seventy-four years of age he wrote to Mr. Eugene Robin in the following terms: 'I am seventy-four years of age, and, being canon of St. Paul's in London, and rector of a parish in the country, my time is equally divided between town and country. I am living amongst the best society in the metropolis, and at ease in my circumstances, in tolerable health, a mild Whig, a tolerating Churchman, and much given to talking, laughing, and noise. I dine with the rich in London, and physic the poor in the country; passing from the sauce of Dives to the sores of Lazarus. I am, upon the whole, a happy man; have found the world an entertaining world, and am thankful to providence for the part allotted to me in it.'" *O si sic omnes.*

